# Evaluation of DNA extraction from granulocytes discarded in the separation medium after isolation of peripheral blood mononuclear cells and plasma from whole blood

**DOI:** 10.1186/1756-0500-6-440

**Published:** 2013-11-01

**Authors:** Janná R Murray, Mangalathu S Rajeevan

**Affiliations:** 1 Division of High-Consequence Pathogens and Pathology, Centers for Disease Control & Prevention, 1600 Clifton Rd Mailstop G41, Atlanta, GA 30333, USA

**Keywords:** DNA extraction, Granulocytes in separation medium, Whole blood

## Abstract

**Background:**

Whole blood is generally processed for plasma and peripheral blood mononuclear cells (PBMCs) from granulocytes/erythrocytes using gradient centrifugation of blood with Histopaue-Ficoll. After separation of plasma and PBMCs, the residual erythrocytes/granulocytes, a rich source of DNA, is often discarded along with the separation medium. In order to isolate DNA from the granulocytes, current methods require the removal of the separation medium and subsequent purification of granulocytes. This report provides a method for extracting DNA using the PAXgene Blood DNA kit from granulocytes without purifying them from the separation medium.

**Findings:**

Based on 719 erythrocyte/granulocyte samples stored frozen for approximately 10 years in Ficoll-Hypaque separation medium, the mean yield of DNA was 395 μg (median = 281 μg; range = 1.36 to 2077.2 μg), with mean A_260_/A_280_ ratio of 1.84 (median = 1.84; range = 1.17 to 2.23). The quality of isolated DNA was sufficient for use as a template for restriction enzyme digestion, real-time PCR, pyrosequencing, and gel based variable number tandem repeats (VNTR) genotyping.

**Conclusions:**

By demonstrating the extraction of substantial amounts of high quality granulocytes DNA without purifying them from the separation medium, this method offers laboratories and biobanks a flexible and cost-effective approach to obtain plasma, PBMCs, and large amounts of DNA from a single blood collection for a variety of molecular genetics/epidemiologic studies.

## Findings

### Background

**Figure 1:**
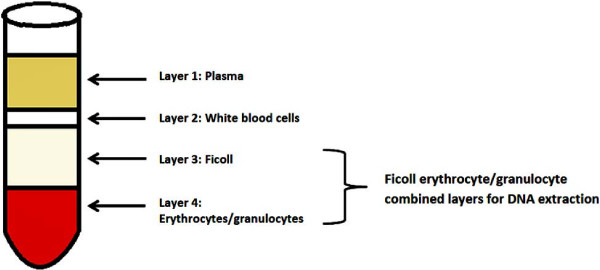
**Diagrammatic representation of granulocytes in separation medium evaluated for DNA extraction in this study.** After centrifugation of diluted whole blood over-layed on Ficoll medium, usually plasma (layer 1) and white blood cells (layer 2) are recovered for further studies. The residual whole blood containing ficoll solution (layer 3) and erythrocytes/granulocytes (layer 4), which is often discarded, was evaluated in this study as a rich source of high quality DNA without further purifications of cells from separation medium.

Whole blood is routinely collected for plasma and PBMC isolation using centrifugation and a density gradient medium such as Ficoll-Hypaque. After centrifugation, whole blood is separated into 4 layers, with plasma at the top followed by white blood cells containing PBMCs, a Ficoll medium layer, and a bottom layer containing erythrocytes and granulocytes (Figure 1) [1]. After the removal of plasma and white blood cells, the remaining Ficoll and the residual erythrocytes/granulocytes, a rich source of genomic DNA for molecular genetic studies, is often discarded. Occasionally, when DNA is required from granulocytes, additional steps to purify them from the separation medium and subsequent DNA extraction steps are needed with the current methods [2-10]. These granulocyte purification steps can include removal of the Ficoll layer, addition of ammonium chloride to the erythrocytes/granulocytes followed by lysis of the erythrocytes, washing, and resuspension of the granulocyte pellet. Further, with the current methods, purification of the granulocytes should be done immediately after blood separation. Here we describe a method that allows for the direct isolation of high quality DNA from stored frozen erythrocytes/granulocytes without purifying them from the separation medium after removal of the plasma and PBMC layers (Figure 1).

### Methods

Centers for Disease Control & Prevention (CDC) Human Subjects Committee approved this study that adhered to the human experimental guidelines of the US Department of Health and Human Services and the Helsinki Declaration. All subjects provided informed consent for the study. Peripheral blood was collected in 8 ml sodium citrate tubes, and PBMCs and plasma fractions were removed using Ficoll [11]. The residual solution containing the Ficoll layer and the erythrocytes/granulocytes was stored in a 50 ml centrifuge tube and frozen at −20°C until DNA extraction (approximately 10 years later). Frozen samples were thawed at 37°C in a water bath and DNA was extracted using the PAXgene Blood DNA Kit (Qiagen, Valencia, CA, USA). The volume of solution containing the separation medium, erythrocytes, and granulocytes was kept constant at 8 ml with addition of phosphate buffered saline solution (pH 7–7.2) as needed. Subsequently, we followed the PAXgene Blood DNA extraction protocol with these samples. DNA concentration and purity were measured using the Nanodrop ND-1000 Spectrophotometer (Thermo Scientific, Wilmington, DE, USA) and the extract was transferred to a 1.5 ml tube and stored at −70°C.

### Results

**Figure 2:**
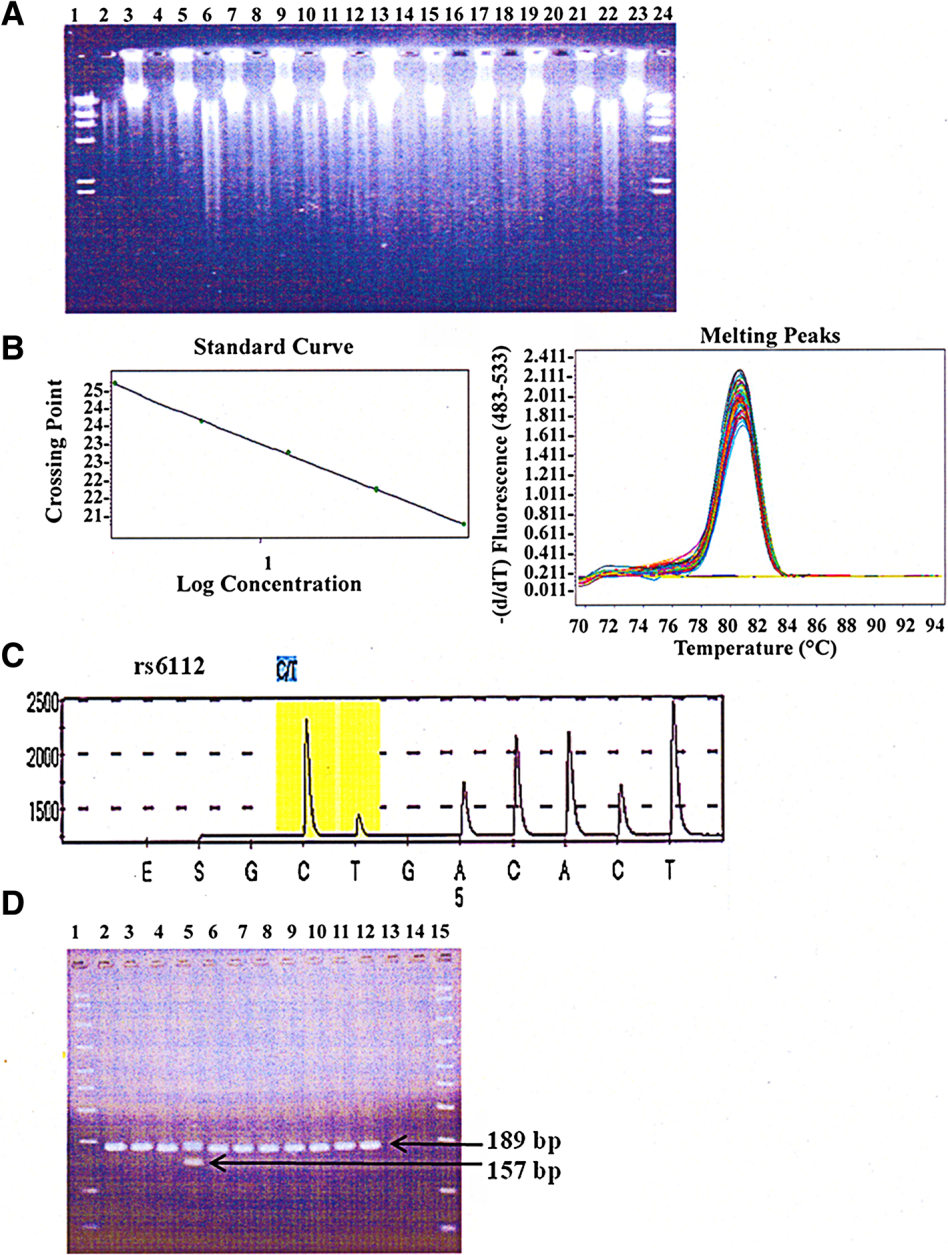
**Evaluation of granulocytes DNA extracted with separation medium after isolation of plasma and PBMCs. (A)** A 0.8% agarose gel stained with ethidium bromide demonstrating high molecular weight DNA, and its near complete digestion with *Eco*RV and *Bam*H1. Lanes 1 and 24 represent the Lambda DNA/*Hin*dIII Marker. Lanes 2–21 are a side by side comparison of the digested and undigested granulocyte DNA extracted with separation medium from 10 subjects. Lanes 22–23 represent digested and undigested whole blood DNA for comparison with granulocyte DNA. **(B)** A typical performance of granulocyte DNA extracted with separation medium in real-time PCR for the *36B4* single copy gene (representative standard curve on the left and melting curves on the right); **(C)** A representative pyrogram for SNP rs6112 using granulocyte DNA extracted with separation medium for successful genotyping; **(D)** Gel-based VNTR analysis of the CCR5 VNTR on granulocyte DNA extracted with separation medium and whole blood DNA. Lanes 1 and 15 represent the 50–2,000 bp markers. Lanes 2–11 represent the granulocyte DNAs extracted with separation medium. Lane 12 represents the whole blood DNA and lanes 13 and 14 contain negative water controls. Arrows indicate expected product sizes (189 bp, and 157 bp) for this VNTR.

In this study, we extracted DNA directly from a total of 719 residual erythrocytes/granulocytes in separation medium after the isolation of plasma and PBMC. The average A_260_/A_280_ ratio was 1.84 (range 1.17 to 2.23; median 1.84) with 99% of the samples being good quality based on A_260_/A_280_ ratio between 1.7 and 2.0. The average yield was 395 μg (range 1.36 to 2077.2 μg; median 281 μg) with 95% of samples yielding >50 μg DNA per sample. Samples yielded high molecular weight DNA as indicated by electrophoresis in 0.8% agarose gel and ethidium bromide stain (Figure 2A). We evaluated 10 DNA samples extracted from granulocytes with separation medium for their suitability for restriction enzyme digestion, real time PCR, pyrosequencing, and VNTR genotyping. DNA from a whole blood sample extracted by the same method was included as a control for these evaluations. We observed a nearly complete digestion of DNA (500 ng) in a 50 μl reaction containing 50 U each of *Eco*RV and *Bam*HI enzymes (New England Biolabs, Ipswich, MA, USA) and incubated at 37°C for 16 hours (Figure 2A). Granulocyte DNA extracted with separation medium was subjected to LightCycler 480 and SYBR Green dye I (Roche Applied Science, Indianapolis, IN USA) based real time PCR for the amplification of *36B4 (also known as RPLPO)* gene using previously published primer sequences [12]. Granulocyte DNA amplified with efficiency (PCR efficiency 1.88) similar to the control whole blood DNA (PCR efficiency 1.89) extracted by the same protocol (Figure 2B). The suitability of granulocyte DNA extracted with separation medium for pyrosequencing was tested by genotyping single nucleotide polymorphism (SNP) rs6112 located in the gene *SERPINA5*. We designed PCR (forward: ACGCTGTACCTGGCAGACACTT and reverse: CGAGGTTCTTAAGCAAGTCCACAA) and sequencing primers (CCTGGCAGACACTTTC) for rs6112 using the Assay Design Software (Qiagen). Pyrosequencing was done with PyroMark PCR Kit (Qiagen) and PSQ 96 MD (Qiagen) following the manufacturer’s instruction. Granulocyte DNA from all 10 samples yielded unambiguous pyrograms and passed automatic genotype calling for rs6112 (Figure 2C). A gel-based assay was used for genotyping of the chemokine receptor 5 (CCR5) VNTR using the PyroMark PCR kit (Qiagen) [13]. PCR bands of expected sizes (157 bp and 189 bp) were seen with all samples (Figure 2D). From these successful results with a variety of enzymatic assays, granulocyte DNA extracted with the separation medium is expected to work equally well with other molecular genetics assays designed to determine copy number variation, methylation status, etc.

### Conclusion

In conclusion, our work demonstrates that DNA can be extracted directly from the residual solution containing the Ficoll layer and erythrocytes/granulocytes left after the isolation of PBMC’s and plasma from whole blood, and that the presence of the separation medium is not a hindrance to extract DNA using the commercially available PAXgene Blood DNA Kit. The quality and quantity of DNA obtained from granulocytes with the separation medium is sufficient enough for a variety of molecular genetics assays. Combining quality and yield, 94.3% (678/719) samples yield >50 μg DNA with an A_260_/A_280_ ratio between 1.7 and 2.0. We recommend that genomic DNA from the remaining small percentage of samples (5.7%) with low yield and low A_260_/A_280_ ratio may be subjected to whole genome amplification if they need to be recovered in certain situations. Unless purified granulocytes are specifically needed as part of study objective, many laboratories often discard the erythrocytes/granulocytes because of the additional steps needed to purify them from the separation medium immediately following the blood separation. Our procedure involves blood collected in sodium citrate tubes, and it will be interesting to compare this method in terms of DNA quality and quantity from residual cells with blood collected in EDTA or heparin coated tubes. In summary, by demonstrating that substantial amounts of high quality DNA can be directly obtained from whole blood residue containing Ficoll solution, erythrocytes, and granulocytes, we have eliminated the need to purify the granulocytes prior to DNA extraction. This method offers laboratories and biobanks a flexible and cost-effective approach to obtain plasma, PBMCs, and large amounts of DNA from a single collection of blood for molecular genetics/epidemiologic studies.

## Competing interests

The authors declare that they have no competing interests.

## Authors’ contributions

JRM carried out laboratory experiments and participated in manuscript writing. MSR as Principle Investigator participated in all aspects of the study and drafting of this manuscript. Both authors read and approved the final manuscript.
